# Cross-Cultural Adaptation and Psychometric Evaluation of the Chinese Version of the Authentic Nurse Leadership Questionnaire

**DOI:** 10.1155/2024/9996979

**Published:** 2024-01-30

**Authors:** Hanyi Wang, Zequan Wang, Cancan Chen, Wanhong Wei

**Affiliations:** ^1^School of Nursing and Health, Zhengzhou University, Zhengzhou 450001, China; ^2^University of Connecticut School of Nursing, 231 Glenbrook Rd, Unit 4026, Storrs, CT, USA; ^3^Department of Nursing, Henan Provincial Key Medicine Laboratory of Nursing, Henan Provincial People's Hospital; Zhengzhou University People's Hospital, Zhengzhou, Henan 450003, China

## Abstract

**Aims:**

To adapt the Authentic Nurse Leadership Questionnaire (ANLQ) to the Chinese cultural context and evaluate its psychometric properties.

**Background:**

Authenticity serves as a pivotal factor in the dynamic interaction between nurse leaders and nurse staff, exerting a profound influence on the growth of nurse individuals, healthcare teams, and organizations. However, there is still a dearth of research instruments to assess nurses' perception of authentic leadership in China.

**Methods:**

After authorization from the original author and technical support had been secured, a systematic process of initial translation, back translation, expert panel review, and pretesting was employed to ensure cross-cultural adaptation in accordance with established guidelines. A two-stage study design was implemented. In stage 1, 189 nurses were sampled for psychometric validation, during which the internal consistency reliability, split-half reliability, and test-retest reliability were tested and exploratory factor analysis was performed. In stage 2, 255 nurses were sampled for confirmatory factor analysis and assessment of convergent and discriminant validity, to further validate the constructs.

**Results:**

In stage 1, the validated instrument showed a Cronbach's alpha value of 0.973, a split-half coefficient of 0.888, and a test-retest reliability coefficient of 0.912. The exploratory factor analysis extracted five dimensions that accounted for 82.629% of the overall variance. The findings in stage 2 showed that the observed data were well fitted to the five-factor theoretical model, with acceptable levels of convergent and discriminant validity.

**Conclusions:**

The Chinese version of the ANLQ demonstrated appropriate psychometric properties, as evidenced by its good reliability and validity. *Implications for Nursing Management*. This study offers nurse administrators and executives a valuable instrument, enabling them to establish leadership evaluation criteria, conduct nurse leader performance appraisals, and assist in selecting new nurse leaders. Ultimately, this contributes to the cultivation and development of exceptional managers capable of providing positive leadership to their followers.

## 1. Introduction

In the rapidly evolving contemporary healthcare setting, ethical dilemmas are frequently encountered [1]. Given the deep integration of nursing with intricate human relationships, these ethical dilemmas are amplified, resulting in undesirable outcomes such as moral distress, decreased job satisfaction, burnout, intrateam conflicts, and compromised patient care [2–4]. Consequently, there is a growing discourse surrounding the importance of values-based leadership and relational leadership as potential strategies to address these dynamic changes [5–7]. Authentic leadership (AL), which is a common component of both aforementioned leadership styles [6, 8, 9], fosters a distinctive relationship between the leader and followers that is marked by high levels of trust, transparency, and integrity [10]. In addition, AL reflects the alignment between the core values of the leader and those demonstrated within the values-based leadership framework [8]. Many studies have emphasized the importance of AL as the foundational basis for various forms of positive leadership [11], highlighting its positive impacts on healthcare staff [6, 12]. These impacts include improving job satisfaction [10], fostering optimism and trust, promoting engagement, and cultivating a supportive work environment [7, 13], ultimately leading to better patient care quality [14]. Furthermore, research has underscored the crucial role of AL in mitigating the emotional exhaustion of followers, reducing workplace stress, alleviating cynicism, preventing burnout, and reducing turnover intention [10, 15–17]. Thus, authenticity, recognized as the paramount aspect of human interaction, is of immense value in healthcare settings that prioritize interpersonal relationships [16] and communication that focuses on the needs and effectiveness of others. Authentic leaders, by exemplifying AL behaviour, not only inspire and motivate their followers while improving their work efficacy [17] but also provide a favourable pathway for their own personal growth and advancement [12].

The majority of existing instruments used to measure AL were developed based on earlier AL theories [11, 18] and showed similarities in terms of their structure, content, and measurement indices. One notable example is the Authentic Leadership Questionnaire (ALQ) developed by Walumbwa et al. [19] in 2008. Notwithstanding the widespread use of the ALQ in healthcare and its adoption in the Chinese nursing domain, it is essential to recognize that the instrument was originally tailored for the for-profit corporate setting. Consequently, the questionnaire may lack fundamental nursing concepts and exhibit limited relevance when applied in the context of nursing practice. In 2011, a group of researchers addressed criticisms of the ALQ and made modifications, resulting in the development of an instrument with four subscales, the 14-item Authentic Leadership Inventory (ALI) [20]. However, the validation process conducted by Davidson et al. [21] for the ALI specifically in the U.S. acute care setting confirmed only a single-factor structure, highlighting the need for further validation of the four-factor structure in the nursing context. Another instrument, the 13-item Authentic Leadership Self-Assessment Questionnaire (ALSAQ, Polish version) [22], demonstrated a favourable Cronbach's alpha of 0.84. However, two out of the three subscales did not meet the criterion of 0.7 for Cronbach's alpha and lacked convergent and discriminant validity [23]. Although the ALSAQ is intended for professional nurses, it assesses registered nurses' perceptions of their own AL, not the AL exhibited by nurse leaders. Moreover, the recruitment of nurses mainly from postgraduate education centres raises doubts about its applicability to clinical settings.

Giordano-Mulligan [24] developed the 29-item Authentic Nurse Leadership Questionnaire (ANLQ) to measure nurses' perception of the AL by nurse leaders, with a specific focus on the core attribute of caring in nursing. By integrating Jean Watson's theory of caring [25] with AL theory, the questionnaire addressed the limitations of existing measures in capturing essential nursing characteristics. The ANLQ has exhibited acceptable psychometric properties with a Cronbach's alpha value of 0.99 and 5 subscales ranging from 0.89 to 0.97. However, its applicability to Chinese nurses from socially and ethnically diverse backgrounds needs to be tested. Therefore, the objective of this study was to report on the cross-cultural adaptation processes and psychometric properties of the ANLQ to prompt a better understanding of its psychological structure in the Chinese cultural context.

## 2. Methods

### 2.1. Translation and Cross-Cultural Adaptation

The ANLQ was translated with the consent and permission of Dr. Giordano-Mulligan. In accordance with Beaton's cross-cultural adaptation guidelines [26] and Brislin's back-translation model [27], several adaptation steps were followed.

#### 2.1.1. Initial Translation with Synthesis

The translation was independently completed by two native Chinese translators, one of whom was a lecturer who had lived in the United States for six years and obtained her doctoral degree in nursing there and the other of whom had graduated with a master's degree and worked as a professional English translator for 14 years. Another professor, a Ph.D. in nursing management, compared the two independent translations word for word and synthesized them into one version.

#### 2.1.2. Back Translation with Reconciliation

The translated version was back-translated to English by two other translators with bilingual backgrounds and no exposure to the original questionnaire, one of whom was a medical doctor who had studied and lived in the United States for 12 years and the other of whom was an American lecturer with no medical background who had worked as a teacher in China for 10 years. Subsequently, another medical doctor who was a visiting scholar at Duke University was invited to compare the two translations and then provided feedback to the original author for verification. Finally, the synthesized Chinese version was modified accordingly to ensure that the expressions retained their original connotation.

#### 2.1.3. Expert Panel Review

Seven experts with master's degrees or above were invited to participate in an expert panel for the study. The panel consisted of a professor with experience in psychological nursing, a methodologist, a linguistics expert, two professors of nursing management, and two nursing administrators with 13 and 34 years of clinical experience. One of the experts, Dr. Sun, was a Chinese American invited by Dr. Giordano-Mulligan to review the translations of the various versions and provide technical support. The cross-cultural adaptation process involved using e-mail, face-to-face interactions, and Zoom videoconferencing, to engage experts in reviewing materials during both initial translation and back translation. The researcher synthesized expert opinions, and in cases of disagreement, the final decision was determined through a written ballot. Subsequently, six experts were invited to assess content validity, scoring each item on a scale from 1 (not relevant) to 4 (highly relevant). A validity threshold was set at an item-level content validity index (I-CVI) of 0.78 or higher and a scale-level average CVI (S-CVI/Ave) of 0.9 or above.

#### 2.1.4. Pilot Testing and Reporting

To ensure that the language used in the questionnaire was both understandable and acceptable, a preliminary investigation was conducted by convenience sampling 30 nurses from various age groups, educational levels, and professional titles. Afterwards, each respondent participated in a 15–20-minute interview to discuss whether they understood each item correctly and if they had any incomprehensible details.

### 2.2. Study Design and Participants

A two-stage cross-sectional survey design employing convenience sampling was implemented in this study. In stage 1, participants were recruited from two tertiary comprehensive hospitals in Henan Province, China. Within each hospital, sample selection was based on the proportion of nurses in different departments to ensure sample diversity. The sample size for the exploratory factor analysis (EFA) adhered to the recommended item response ratio of 1 : 5 to 1 : 10 [28]. To accommodate a potential 20% questionnaire invalidity rate, a minimum sample size of 182 participants was determined. The inclusion criteria were registered nurses who (1) were employed and age 18 years or older, (2) had worked with their current nurse leader for ≥1 year, and (3) were under the direct leadership of the nurse leader. The exclusion criteria were nurses who were not at work (e.g., on further training, missions, or leave of absence) and those rotating within the department during the study period. For the test-retest reliability assessment, thirty respondents were selected and returned to complete the C-ANLQ after a two-week interval.

Moving to stage 2, participants were drawn from the other two tertiary comprehensive hospitals ensuring a distinct selection from those involved in stage 1. It is generally accepted that the sample size for confirmatory factor analysis (CFA) should not be less than 200 [29], with an additional 20% accounting for potential invalid responses, thus establishing a minimum sample size of 250 participants. Inclusion criteria mirrored those of stage 1 participants.

### 2.3. Instruments

#### 2.3.1. General Information Questionnaire

The self-designed demographic questionnaire included 8 questions on the participant's age, gender, marital status, department affiliation, years of experience, years working with the current nurse leader, education, and professional title.

#### 2.3.2. Authentic Nurse Leadership Questionnaire

The ANLQ is a 29-item instrument developed by Giordano-Mulligan [24] with five subscales: self-awareness, moral ethical courage, relational integrality, shared decision making, and caring. The instrument uses a 5-point Likert scale ranging from 0 (never) to 4 (all time), with an overall score ranging from 0 to 116. A higher score indicates a higher level of perceived authentic leadership by nurses. The instrument was validated by Hwang et al. in a group of Korean nurses [15], with a total Cronbach's alpha coefficient of 0.97, which indicated good psychometric properties.

#### 2.3.3. Authentic Leadership Questionnaire

The 16-item ALQ was developed by Walumbwa et al. [19] and consists of four subscales: self-awareness, relational transparency, internalized moral perspective, and balanced processing. The items are measured on a 5-point Likert scale from 0 (highly disagree) to 4 (fully agree), with a total score ranging from 0 to 64. The Cronbach's alpha coefficient for the Chinese version [30] of ALQ was 0.95, and the one in this study was also 0.95.

### 2.4. Data Collection

Initial support was secured from the participating hospitals and nursing supervisors before commencing the field survey. The data for stage 1 were gathered between January and February 2023. Participants received printed questionnaires in sealed envelopes from the investigator and submitted them on the spot. To minimize bias, they completed the questionnaire in the absence of the nurse leader. Additionally, for those willing to participate in the follow-up survey, we recorded their contact details and assigned numbers for the assessment of test-retest reliability two weeks after the initial survey. Stage 2 data collection occurred between March and April 2023, following the same protocol as stage 1.

### 2.5. Data Analysis

The data analysis was conducted using IBM SPSS software (version 26.0) and AMOS software (version 24.0) for Windows. Descriptive statistics were used to analyse participant characteristics as necessary. In stage 1, the internal reliability of the scale was assessed using both Cronbach's alpha coefficient and the split-half reliability coefficient. A Cronbach's alpha coefficient of ≥0.9 was considered excellent, ≥0.8 indicated high reliability, and ≥0.7 was considered acceptable. The Spearman–Brown coefficient was used to correct the number of items in which the two subscales were not equal [28]. Test-retest reliability was used to capture external reliability, and a value greater than 0.7 indicated a high degree of stability of the scale. Item analysis was conducted with the critical ratio (CR) method (a sample *t*-test to compare the differences between the upper 27% and lower 27% of the subgroups), and homogeneity tests were used as screening indicators for each of the individual items. Items with a CR value <3.0, item-total correlation coefficient <0.4, or an increase of 0.5 or more in Cronbach's alpha after the deletion of the item were removed.

Construct validity was verified by a joint evaluation of EFA and CFA. The Kaiser‒Meyer‒Olkin (KMO) test and Bartlett's test of sphericity were initially performed to determine the sample fit to confirm that EFA was appropriate. Factors were extracted based on an eigenvalue >1.0, scree plot, and factor loading ≥0.5. In stage 2, the CFA further validated the default model from stage 1, in which the parameters were estimated using the maximum likelihood method. Convergent validity was considered appropriate with an average variance extracted (AVE) >0.5, standardized factor loading >0.5, and composite reliability >0.7 [31]. Determining discriminant validity, we ensured that the square root of the AVE was greater than the correlation coefficient between each factor and the other factors. Pearson correlation analysis was used to determine the association between the C-ANLQ and the ALQ to evaluate criterion validity.

## 3. Results

### 3.1. Translation and Cross-Cultural Adaptation

In the cross-cultural adaptation phase, considering the potential difficulty of Chinese nurses comprehending the term “visible” in item 4, the word was replaced to convey a meaning understandable in the Chinese healthcare context. Following two rounds of expert discussions, “visible” was translated as “appearing frequently in front of nurses and actively interacting with them.” Additionally, after consulting with the original author and expert panel, item 22 was modified to read “My nurse leader participates in organizations or activities associated with the nursing profession,” providing a more detailed explanation for nurses. This phase involved revising a total of 12 items and adjusting the labels for both dimensions and the questionnaire title.

During the pilot testing phase, nurses took approximately 3–5 minutes to complete the questionnaire. In response to nurses' misunderstandings of “group pressure,” the wording was amended, and supplementary instructions were included based on integrated group feedback. Consequently, item 9, “My nurse leader would not be influenced by negative group pressure,” was revised to “My nurse leader would not be influenced by negative group pressure (e.g., the influence of doctors, nurses, supervisors, medical techniques and colleagues in clinical support departments).” Item 28, “My nurse leader pampers the personal growth of followers,” was adjusted to “My nurse leader fosters followers and promotes their personal growth.” This change was made because several nurses noted that “pampering followers” behaviour was slightly exaggerated and that it was more appropriate to use the language of fostering between leaders and followers. Additionally, four redundant statements were streamlined during the pretest. Throughout the research process leading to this stage, the Chinese version of the ANLQ was formed, proving relevant to clinical nursing work and easily comprehensible.

### 3.2. Participant Characteristics

In stage 1, all 200 participants who met the criteria responded and returned the questionnaires. However, 11 questionnaires were found to be spurious, as all the items were filled in with the same answer. Consequently, 189 questionnaires (94.50%, 189/200) were deemed valid. Moving to stage 2, the survey involved 280 registered nurses, 278 of whom returned the questionnaires, yielding a response rate of 99.29%. After excluding incomplete questionnaires and spurious responses, a total of 255 questionnaires (91.07%, 255/280) were considered valid. The characteristics and demographics of the participants in the two stages are shown in Table 1.

### 3.3. Reliability and Item Analysis

The item-total correlations for the 29-item C-ANLQ ranged from 0.600 to 0.875, indicating that item-total correlations not only achieved significance (*p* < 0.001) but also showed a mid-high correlation (*r* > 0.40). The independent *t*-test showed that the CR value for each item significantly differed (*p* < 0.001) in the high subgroup (>73%, score = 92) and the low subgroup (<27%, score = 68). Consequently, there was no need to delete any items, since they were sufficiently distinct from one another, and there was a good homogeneity from the items to the total scale.

Cronbach's alpha coefficient and the split-half reliability were used to validate the internal consistency of the C-ANLQ. The Cronbach's alpha value was 0.973 for the total scale and ranged from 0.921 to 0.972 for the subscales, with a split-half reliability of 0.888. The alpha coefficients ranged from 0.971 to 0.973 if an item was deleted, indicating that no item needed to be considered for deletion. Moreover, there was a statistically significant correlation between the test and retest for “self-awareness” (*r* = 0.769, *p* < 0.001), “moral ethical courage” (*r* = 0.818, *p* < 0.001), “relational integrality” (*r* = 0.850, *p* < 0.001), “shared decision making” (*r* = 0.814, *p* < 0.001), “caring” (*r* = 0.878, *p* < 0.001), and the C-ANLQ total score (*r* = 0.912, *p* < 0.001).

### 3.4. Content Validity and Criterion Validity

Six experts were invited to rate the content validity two weeks after the expert panel review. The results showed that the I-CVI ranged from 0.83 to 1.00 (greater than 0.78), and the S-CVI/Ave was 0.97 (greater than 0.9), indicating good content validity. The Pearson's correlation results (see Table 2) showed positive correlations of the C-ANLQ (and subscales) with both the C-ALQ (and subscales), from 0.586 to 0.783, and all correlations were significant (*p* < 0.001).

### 3.5. Construct Validity

**Figure 1:**
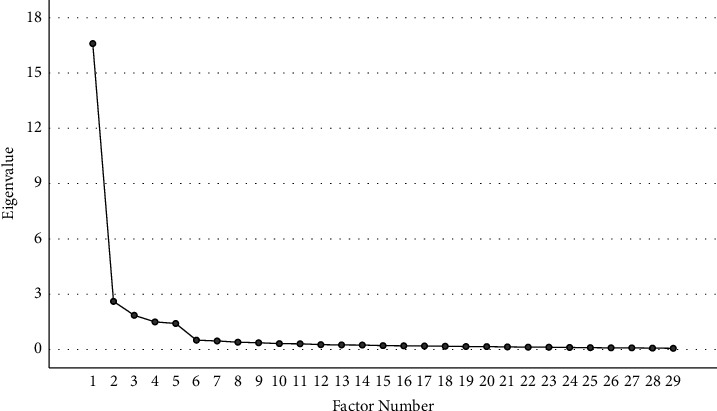
Scree plot.

The KMO coefficient was 0.954, and Bartlett's test was significant (chi-square = 6450.072, *p* < 0.001), which supported the feasibility of EFA (with a sample of 189 nurses, stage 1). Five factors were extracted (see Figure 1) by using principal component analysis (PCA) with varimax rotation; these factors explained 82.629% of the total variance.  Table 3 details the results of descriptive statistical analysis and EFA for the C-ANLQ. The factor loadings on each item exceeded the acceptable level in the range of 0.697 to 0.863, and no cross-loading was observed, with the communality of each item over 0.4; thus, all items were retained.

**Figure 2:**
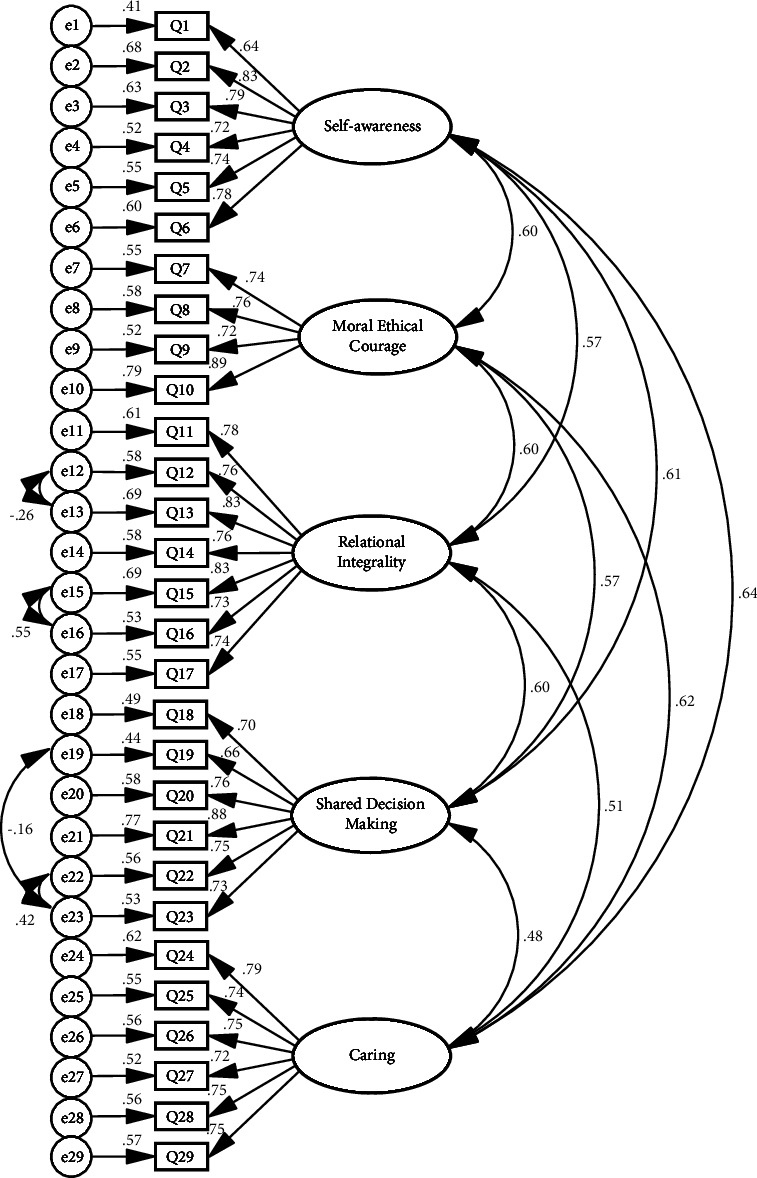
Confirmatory factor analysis modified model.

The five-factor structure was then validated using CFA on a sample of 255 nurses in stage 2. The initial model fit indices showed an inadequate model fit, which was corrected by adding four residual paths based on the principle of maximizing the modification index [32], which ultimately improved the model fit and formed the CFA-modified model (see Figure 2). The initial and modified model fit indices are shown in Table 4.

### 3.6. Convergent and Discriminant Validity

The standardized factor loadings of all items in the C-ANLQ were statistically significant (*p* < 0.001) and higher than 0.5, ranging from 0.637 to 0.891. In addition, the AVE estimates ranged from 0.560 to 0.608, all of which were higher than 0.5, while the composite reliability estimates ranged from 0.884 to 0.914, with all above 0.7. As shown in Table 5, the square roots of the AVE of all five dimensions were greater than their correlation coefficients, indicating that the dimensions were discriminated.

## 4. Discussion

An integral part of nursing practice is the nurse leader's ability to build authentic relationships with nurse staff through his or her authentic leadership. This ability generates positive attitudes among nurse staff, which, in turn, improves the quality of care. This study describes the first verified study of the C-ANLQ in China, which was validated by a two-stage survey. The adapting process of the instrument followed Beaton's cross-cultural adaptation guidelines [26] and Brislin's back-translation model [27]. These guidelines suggest that in cross-cultural research, it is crucial to not only maintain semantic and conceptual equivalence with the original scale but also paraphrase one's own culture-specific experiences or behaviours. This ensures the relevance and applicability of the instrument in the specific cultural context. Moreover, continuous communication with the original author was pivotal to ensure that the instrument was suitable for the Chinese cultural context while preserving the integrity of the original version.

The C-ANLQ demonstrated good psychometric properties in the sample of registered nurses in this study. For item analysis, the item-total correlations were acceptable, as all were over 0.4, indicating that all items in the instrument measured the same constructs. In addition, all 29 items had CR values in excess of 3 and at the 0.05 level of significance, showing that all items were able to discriminate between the levels of variance reflected by different respondents. In the study, Cronbach's *α* coefficient for the total scale and each subscale exceeded 0.9, representing a favourable level of internal consistency reliability, which is compatible with the results of other studies [15, 33]. The Cronbach's *α* coefficient derived after deleting each item separately never surpassed the overall Cronbach's *α* coefficient of the questionnaire, indicating that the psychological traits to be measured by each item in the questionnaire were consistent. In the retest after a two-week interval, the test-retest reliability of the five factors was between 0.769 and 0.878, which was consistent with the Giordano-Mulligan results [24] between 0.780 and 0.880, showing good measurement stability.

This study carried out seven iterations of EFA, resulting in the extraction of five factors: self-awareness, moral ethical courage, relational integrality, shared decision making, and caring. These factors collectively accounted for 82.629% of the total variance of the questionnaire, and the factor loading of each item exceeded 0.4, indicating consistency with the original instrument structure. However, the initial predefined model did not achieve the desired fit during CFA, possibly due to correlations between items [34]. Consequently, the model underwent a progressive revision based on correction index suggestions, including the addition of four residual paths to improve statistical fitness. The four residual paths added indicate that certain items could not be fully explained by their respective latent variables. For example, the residual paths between item 23 and item 19 and between item 23 and item 22 indicate associations beyond the latent variables of “shared decision making” in the work, possibly involving other factors. This variation may be attributed to cultural differences in the interpretation of the questionnaire items. In individualistic cultures such as the United States, personal viewpoints and active participation in management decision making are valued, whereas collectivistic cultures such as China emphasize deference to leadership decisions [35, 36]. Additionally, the presence of residual paths between item 12 and item 13 and between item 15 and item 16 suggested the involvement of additional shared factors beyond “relational integrality.” This may be due to the proficiency of Chinese nurse staff in building relationships, as they are adept at quickly establishing strong personal connections with leaders and cultivating common interests [37], thus experiencing greater influence from nurse leaders. The modified model incorporates these residual paths, offering a more accurate representation of the structure of the sample data. The modified chi-square degrees-of-freedom ratio of 1.131 meets the stricter fit criterion of less than 2 and surpasses the Giordano-Mulligan index results [24]. Although the adjusted goodness-of-fit index (AGFI) did not reach the optimal fit index, probably due to the limitation of the sample size, it was close to 0.9, which remained acceptable. AGFI is calculated from the goodness-of-fit index (GFI) and is generally less than the estimate of GFI [34]. In this study, the GFI met the criteria, and the other fit indicators met the needed standard. Therefore, in general, the modified five-factor model was in good fit with the sample data and matched the theoretical design of the original instrument.

In terms of content validity, the expert base information, qualification, and consultation process ensured the validity of the CVI evaluation. Following a single round of expert consultation, the C-ANLQ met the criteria of S-CVI/Ave >0.90 and I-CVI >0.78, demonstrating its good content validity. The scores between the four dimensions of the C-ANLQ and the four dimensions of the ALQ showed a significant positive correlation, aligning with the findings of Giordano-Mulligan [24]. Furthermore, a newly identified factor, “caring,” pertains to authentic leaders' willingness to serve others and demonstrate concern for the well-being of their followers [38]. Such leaders possess self-transcendent values and exhibit heightened levels of compassion. In this study, caring exhibited significant positive correlations with the total ALQ score and its four dimensions, with correlation coefficients of 0.696, 0.661, 0.663, 0.586, and 0.641 (all *p* < 0.001). This may be attributed to the fact that nursing, as a caring profession, plays a crucial role in the perception of AL from nurse leaders. This statement is in line with the ANLQ conceptual framework [38] in which caring is a natural attribute. Therefore, these results showed a promising correlation between the C-ANLQ and ALQ, with good criterion validity.

Regarding convergent validity, all five latent variables exhibited a composite reliability greater than 0.7, which determined the good inherent quality of the model. The AVE exceeded 0.5, suggesting that the latent construct accounted for at least 50% of the indicator variance [34]. Additionally, the standardized factor loading of each item surpassed 0.5, indicating a good model fit [31]. Collectively, these findings support the strong convergent validity of the latent construct. For discriminant validity, the square root of the AVE for each of the five dimensions exceeded the correlation coefficient between that dimension and the others. This observation indicates good discriminability among the five dimensions and validates the presence of distinct constructs within the C-ANLQ. Therefore, the C-ANLQ can be deemed to possess five dimensions with reasonable discriminant validity.

### 4.1. Limitations

The survey conducted in this study was limited to tertiary comprehensive hospitals in Henan Province, China, using a convenience sampling method, which might restrict the generalizability of the findings. Future research could encompass a broader range of participants, such as outpatient nurses and those working in sterilization supply centres, and validate the instrument in diverse geographic regions across China. While the C-ANLQ was administered by the researcher in person to minimize the influence of nurse leaders, response bias might still exist due to social expectations, potentially inflating the response scores compared to actual internal scores.

## 5. Conclusion

This study adhered to systematic guidelines to translate the English version of the ANLQ into Chinese, establishing an expert panel to conduct cross-cultural adaptation and language validation within a Chinese nursing context. The C-ANLQ was subsequently verified as a valid instrument, exhibiting satisfactory reliability and validity through a two-stage survey involving registered nurses.

### 5.1. Implications for Nursing Management

Nurse leaders hold pivotal roles as primary managers and direct supervisors of nurse staff, exerting a significant influence on work attitudes and behaviours. This study offers nursing administrators a novel and contextually appropriate assessment instrument, the C-ANLQ, to measure nurses' perception of AL, with far-reaching implications for healthcare organizations aiming to foster AL development among nurse leaders. The C-ANLQ serves as a benchmark for nurse administrators and executives when establishing leadership evaluation criteria, designing training programs, selecting new nurse leaders, and conducting performance appraisals. Furthermore, a longitudinal perspective on the enduring impact and growth trajectory of nurse leaders' AL in practice provides valuable insights into the evolution of AL behaviours over time.

## Figures and Tables

**Table 1:** Social and demographic information of the participants (stage 1, *n* = 189; stage 2, *n* = 255).

Descriptive characteristics	Stage 1		Stage 2	
	Frequency (*n*)	Percentage (%)	Frequency (*n*)	Percentage (%)
Gender				
Male	18	9.5	17	6.7
Female	171	90.5	238	93.3
Age, years (M ± SD)	32.40	6.415	31.47	6.526
Education				
High school	4	2.1	5	2.0
Junior college	39	20.6	40	15.7
Undergraduate	145	76.7	201	78.8
Postgraduate or above	1	0.5	9	3.5
Marital status				
Unmarried	45	23.8	93	36.5
Married	141	74.6	159	62.4
Divorced or widowed	3	1.6	3	1.2
Department affiliation				
Internal medicine	49	25.9	84	32.9
Surgery	22	11.6	59	23.1
Emergency department	16	8.5	29	11.4
Intensive care unit	36	19.0	24	9.4
Obstetrics and gynaecology	12	6.3	22	8.6
Paediatrics	16	8.5	23	9.0
Operating room	38	20.1	14	5.5
Work experience, years (M ± SD)	9.96	7.457	9.39	6.515
Work with the current nurse leader, years (M ± SD)	6.51	5.249	5.32	4.407
Professional title				
Primary nurse	39	20.6	43	16.9
Nurse practitioner	66	34.9	109	42.7
Nurse-in-charge	83	43.9	99	38.8
Deputy director nurse	1	0.5	4	1.6

M ± SD: mean ± standard deviation.

**Table 2:** Pearson's correlations between C-ANLQ and ALQ (*n* = 40).

	ALQ (total)	Self-awareness	Internalized moral perspective	Relational transparency	Balanced processing
C-ANLQ (total)	0.783	0.733	0.740	0.697	0.705
Self-awareness	0.774	0.755	0.727	0.675	0.689
Moral ethical courage	0.690	0.620	0.673	0.650	0.591
Relational integrality	0.778	0.728	0.725	0.735	0.676
Shared decision making	0.740	0.684	0.688	0.633	0.708
Caring	0.696	0.661	0.663	0.586	0.641

All *p* < 0.001.

**Table 3:** Results of descriptive statistics and exploratory factor analysis (*n* = 189).

Items	Mean	SD	Factor					*C* ^2^
			1	2	3	4	5	
Q1	3.05	0.849	0.215	0.123	0.149	**0.756**	0.177	0.686
Q2	2.74	0.925	0.224	0.256	0.289	**0.740**	0.138	0.766
Q3	2.70	0.994	0.215	0.199	0.303	**0.734**	0.218	0.764
Q4	2.84	0.928	0.211	0.286	0.374	**0.697**	0.127	0.768
Q5	3.17	0.794	0.204	0.152	0.057	**0.730**	0.307	0.696
Q6	2.97	0.841	0.128	0.194	0.152	**0.781**	0.242	0.746
Q7	2.38	0.923	0.261	0.116	0.188	0.306	**0.813**	0.873
Q8	2.39	1.039	0.262	0.152	0.264	0.259	**0.798**	0.865
Q9	2.16	1.072	0.184	0.075	0.172	0.193	**0.863**	0.850
Q10	2.14	1.088	0.185	0.157	0.137	0.241	**0.837**	0.836
Q11	2.49	1.133	**0.758**	0.291	0.225	0.234	0.111	0.778
Q12	2.82	0.984	**0.722**	0.232	0.312	0.289	0.274	0.832
Q13	2.70	1.010	**0.755**	0.323	0.258	0.211	0.273	0.860
Q14	2.53	1.065	**0.752**	0.243	0.187	0.169	0.157	0.712
Q15	2.74	0.947	**0.799**	0.262	0.190	0.165	0.234	0.825
Q16	2.74	0.964	**0.820**	0.217	0.283	0.157	0.217	0.871
Q17	2.63	1.076	**0.709**	0.282	0.337	0.301	0.137	0.806
Q18	2.77	1.076	0.337	**0.784**	0.281	0.209	0.035	0.851
Q19	2.96	0.880	0.253	**0.823**	0.241	0.247	0.082	0.866
Q20	2.78	1.054	0.285	**0.819**	0.275	0.198	0.055	0.870
Q21	2.79	0.977	0.268	**0.817**	0.269	0.166	0.142	0.860
Q22	2.93	0.954	0.237	**0.819**	0.184	0.204	0.181	0.836
Q23	2.87	0.981	0.213	**0.803**	0.300	0.184	0.195	0.851
Q24	2.51	1.070	0.276	0.296	**0.770**	0.261	0.201	0.865
Q25	2.66	1.038	0.293	0.423	**0.703**	0.295	0.210	0.890
Q26	2.60	1.045	0.247	0.360	**0.752**	0.234	0.244	0.870
Q27	2.52	1.019	0.277	0.280	**0.812**	0.245	0.207	0.917
Q28	2.53	0.998	0.335	0.253	**0.789**	0.215	0.180	0.876
Q29	2.47	1.034	0.331	0.301	**0.781**	0.213	0.143	0.875
Eigenvalues			16.599	2.608	1.852	1.495	1.409	
Explained variance (%)			57.239	8.992	6.386	5.154	4.859	
Cumulative variance (%)			57.239	66.230	72.616	77.770	82.629	
Cronbach's alpha of each subscale			0.957	0.963	0.972	0.921	0.941	

F1: relational integrality; F2: shared decision making; F3: caring; F4: self-awareness; F5: moral ethical courage; SD: standard deviation; *C*^2^: communality. The factor loading values with absolute values greater than 0.400 are shown in bold.

**Table 4:** Model fit indices of the five-factor model in confirmatory factor analysis.

	*χ* ^2^/*df*	GFI	AGFI	RMSEA	NFI	TLI	CFI	PGFI
Initial model	1.423	0.879	0.857	0.041	0.889	0.960	0.964	0.742
Modified model	1.104	0.905	0.887	0.020	0.915	0.990	0.991	0.756
Target value	<2.0	>0.90	>0.90	<0.05	>0.90	>0.90	>0.90	>0.50

GFI: goodness-of-fit index; AGFI: adjusted goodness-of-fit index; RMSEA: root mean square error of approximation; NFI: normed fit index; TLI: tacker-Lewis index; CFI: comparative fit index; and PGFI: parsimony goodness-of-fit index.

**Table 5:** Discriminant validity (*n* = 255).

	SA	MEC	RI	SDM	Caring
SA	**0.751**				
MEC	0.600^*∗*^	**0.780**			
RI	0.567^*∗*^	0.600^*∗*^	**0.777**		
SDM	0.614^*∗*^	0.570^*∗*^	0.604^*∗*^	**0.749**	
Caring	0.641^*∗*^	0.616^*∗*^	0.513^*∗*^	0.478^*∗*^	**0.750**
AVE	0.564	0.608	0.604	0.560	0.563

^*∗*^ *p* < 0.001; AVE, average variance extracted; SA: self-awareness; MEC: moral ethical courage; RI: relational integrality; SDM: shared decisionmaking. Bold values show the AVE square root in each of the five dimensions.

## Data Availability

The data supporting the results of this study are available from the corresponding author upon reasonable request.
